# Thin Patient, Fatty Liver

**DOI:** 10.7759/cureus.4139

**Published:** 2019-02-26

**Authors:** V V Pavan Kedar Mukthinuthalapati, Bashar M Attar, Yazan Abu Omar, Vikas Nath, Carol Czapar, Seema R Gandhi

**Affiliations:** 1 Internal Medicine, John H. Stroger Jr. Hospital of Cook County, Chicago, USA; 2 Gastroenterology and Hepatology, John H. Stroger Jr. Hospital of Cook County, Chicago, USA; 3 Pathology, John H. Stroger Jr. Hospital of Cook County, Chicago, USA

**Keywords:** non alcoholic steatohepatitis, myositis

## Abstract

A 49-year-old lady with no past medical history presented with dysphagia and 40-pound weight loss, which occurred over eight months. On physical examination, she had proximal muscle weakness and crackles in basilar regions of the lungs. Labs were significant for low albumin, elevated transaminases, and high aldolase. Imaging suggested aspiration pneumonitis in both lungs and hepatic steatosis. A swallow evaluation revealed oropharyngeal dysphagia and muscle biopsy confirmed a rare form of myositis. A liver biopsy showed steatohepatitis and a diagnosis of starvation-induced steatohepatitis was made. The patient succumbed to hypoxic respiratory failure from aspiration pneumonitis before the treatment for myositis could be initiated. We report the first case of starvation-induced steatohepatitis in a patient with dysphagia from myositis affecting the oropharyngeal musculature.

## Introduction

Nonalcoholic fatty liver disease (NAFLD) affects 20% - 30% of the adult population in the United States [1]. NAFLD is typically found in patients with obesity, diabetes mellitus, and hyperlipidemia. The spectrum of NAFLD ranges from isolated hepatic steatohepatitis that can lead to liver cirrhosis [1-2]. Most of the cases of NAFLD are associated with metabolic syndrome and its risk factors, and less commonly related to drugs [2]. We present an unusual case of NAFLD secondary to starvation from neuromuscular dysphagia caused by a rare inflammatory myopathy, that progressed to cirrhosis.

## Case presentation

A 49-year-old Hispanic female with no significant past medical history presented to the emergency department with progressive dysphagia to liquids and solids and 40-pound unintentional weight loss over the last eight months. She denied a history of alcohol abuse, herbals, supplements or environmental exposures. Upon presentation, blood pressure was 99/57 mmHg and the pulse rate was 122/minute. On examination, she was cachectic, had 4 to 4+ power in all extremities, bilateral wrist swelling, bi-basilar crackles, and bilateral pedal edema. Her body mass index (BMI) was 22; her BMI one year ago was 30. Liver enzymes, a year prior to the presentation, were normal.

**Figure 1:**
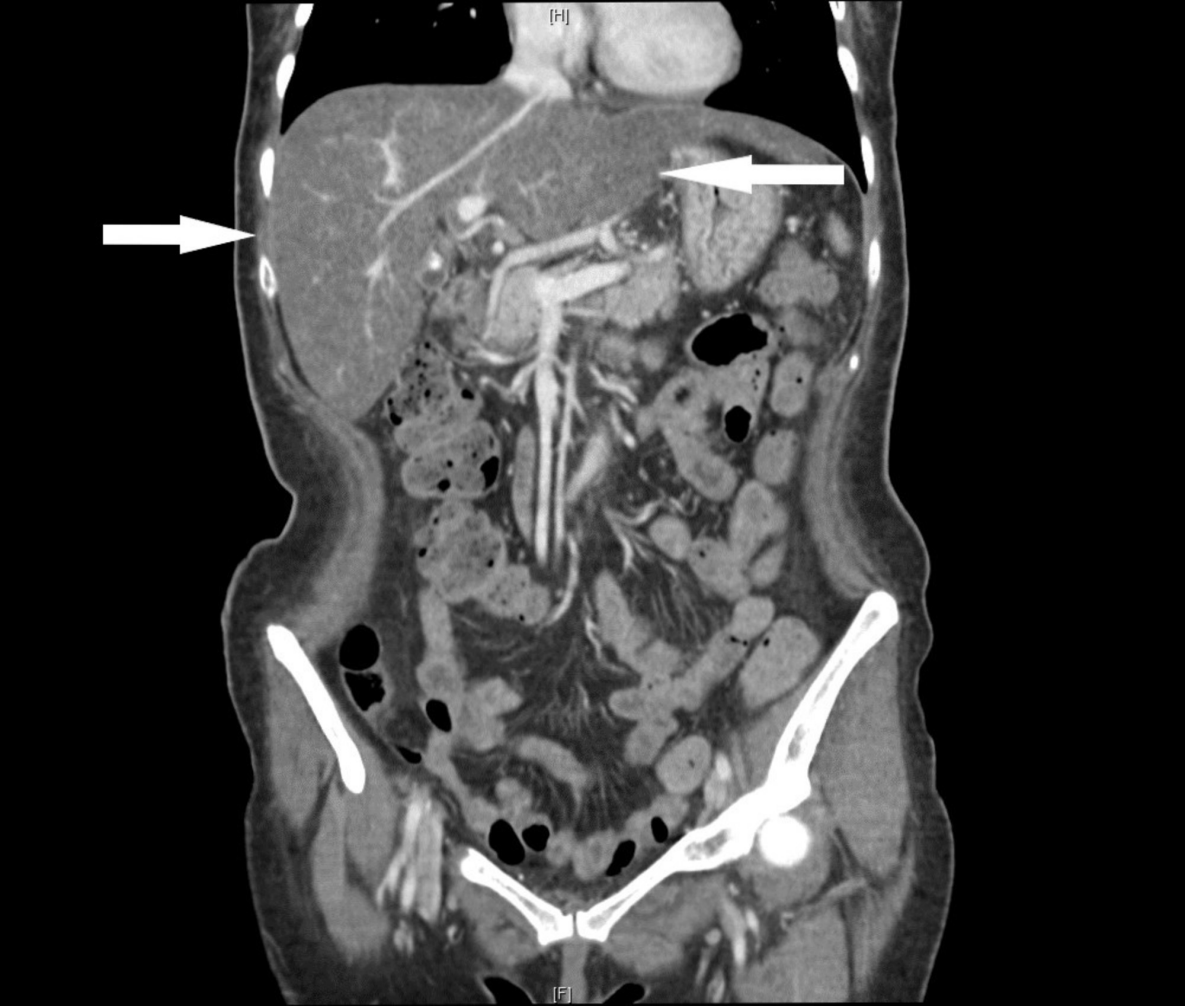
Computed tomography (CT) of the abdomen and pelvis with intravenous contrast on admission, showing hepatomegaly (marked by arrows)

**Figure 2:**
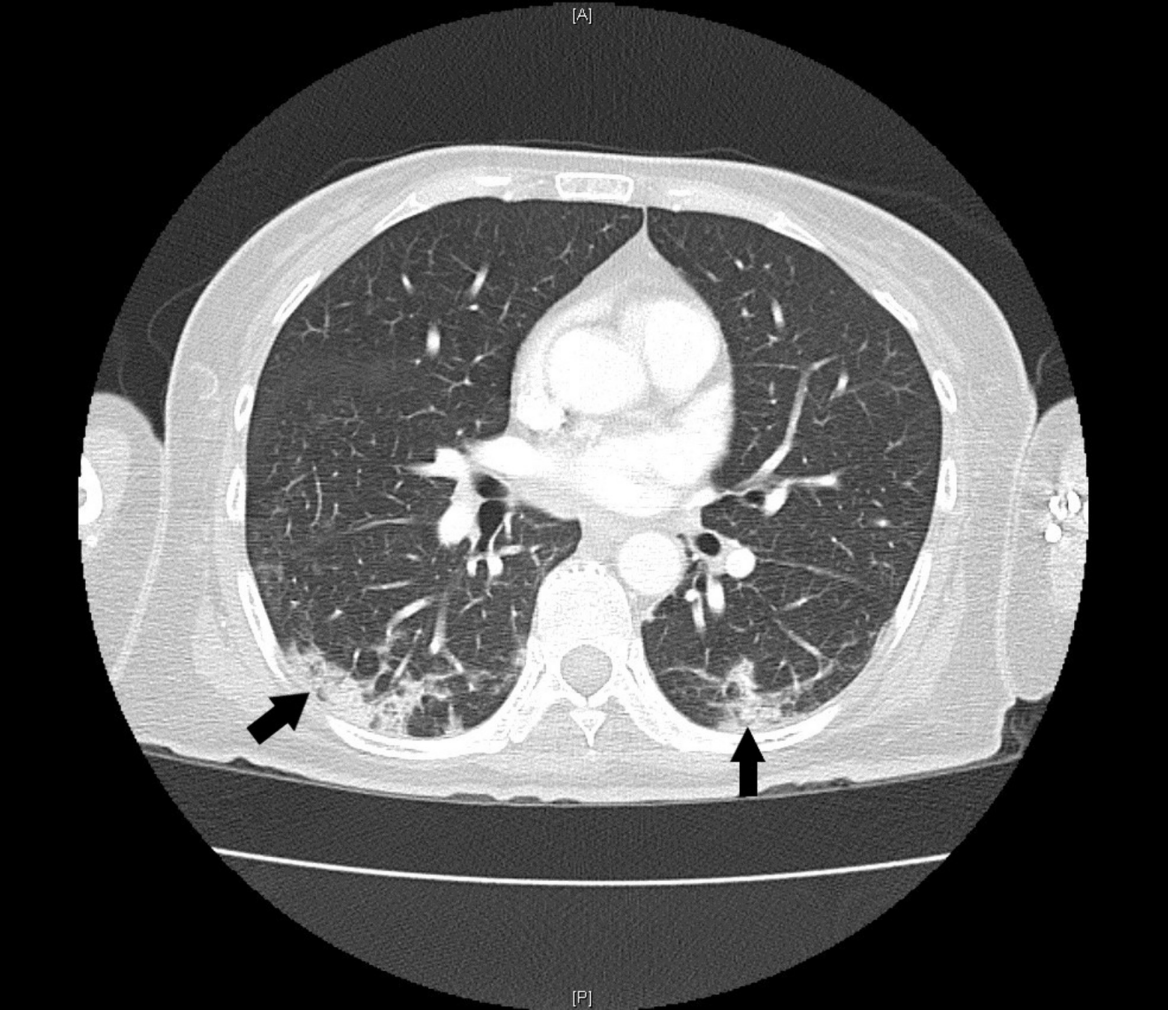
Computed tomography (CT) of the chest with intravenous contrast showing infiltrates in dependent portions of the lung (marked by arrows)

**Figure 3:**
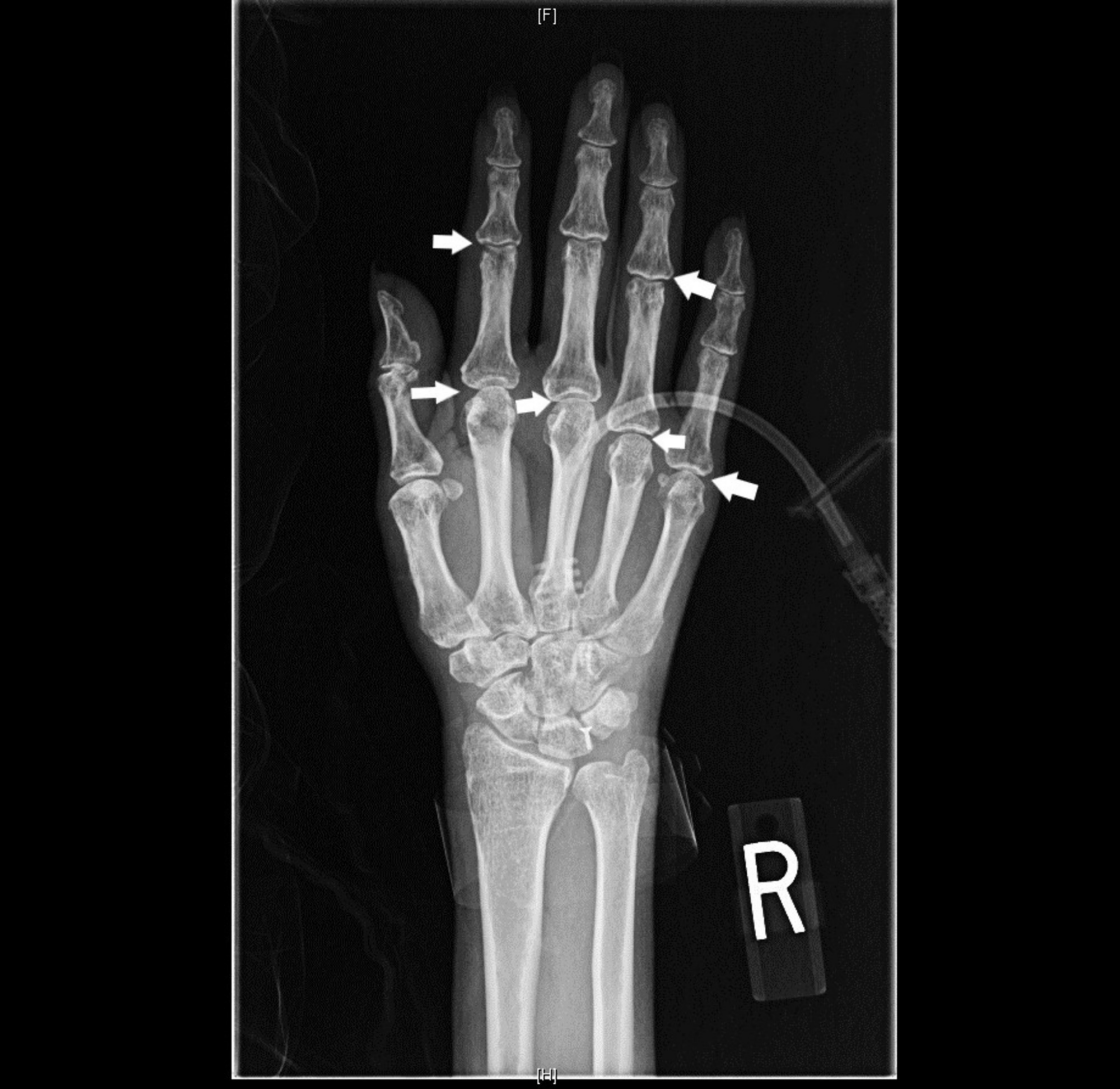
X-ray of the hand showing juxta-articular osteopenia (marked by arrows)

Labs were significant for blood urea nitrogen 7 mg/dL, creatinine 0.3 mg/dL, albumin 1.6 g/dL, total bilirubin 1.2 mg/dL, direct bilirubin 0.9 mg/dL, alkaline phosphatase 722 units/L, gamma-glutamyl transferase 958 units/L, aspartate aminotransferase 325 units/L, alanine aminotransferase 82 units/L, hemoglobin 10.3 g/dL, ferritin 2468 ng/mL, transferrin saturation 85%. Her creatine kinase (CK) was 55 units/L (normal range 0-163), aldolase 10.4 units/L (normal range < 8.1) and C-reactive protein was 1.71 mg/dL (normal range < 0.6). Antinuclear antibody (ANA), anti-Jo-1, and anti-topoisomerase I antibody were negative. There were no documented liver function tests prior to presentation. Computed tomography (CT) of the chest, abdomen, and pelvis revealed ground glass opacities involving bilateral lung apices and dependent portions of the lower lobes, consistent with aspiration pneumonia, and hepatomegaly with hepatic steatosis (Figures 1-2). Anti-mitochondrial antibody assay, HFE gene mutation analysis, ceruloplasmin, viral hepatitis panel, alpha-1 antitrypsin level and anti-smooth muscle antibody assay were sent to evaluate the elevated liver enzymes and were negative. Magnetic resonance cholangiopancreatography (MRCP) did not reveal any biliary pathology. A bedside swallow evaluation revealed oropharyngeal dysphagia and X-ray of the hands revealed juxta-articular osteopenia (Figure 3).

**Figure 4:**
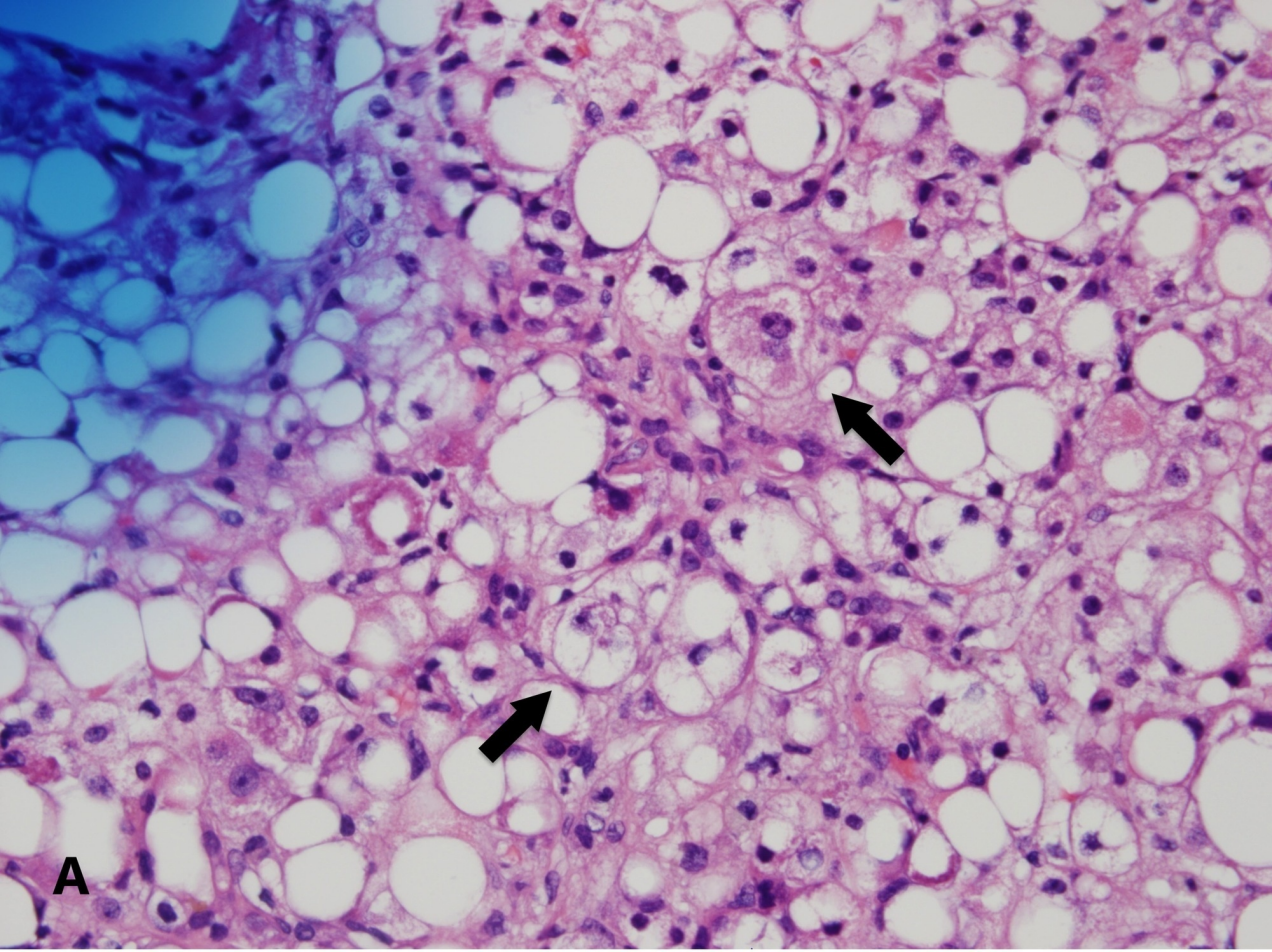
Pathology slide of the liver biopsy showing steatohepatitis and Mallory bodies within ballooning degeneration (marked by arrows)

**Figure 5:**
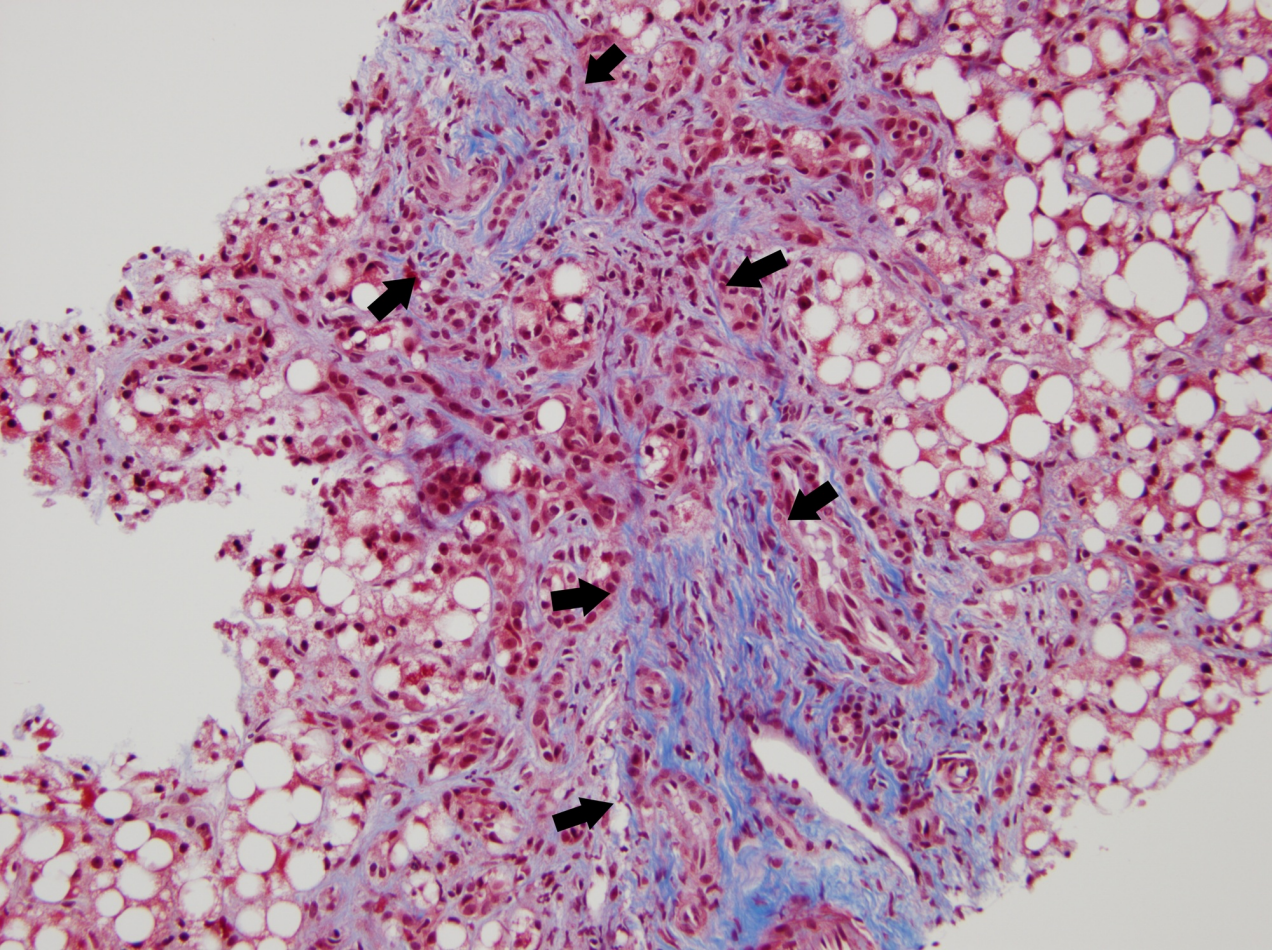
Trichrome stain of the liver pathology slide showing F3 fibrosis (between the arrows)

**Figure 6:**
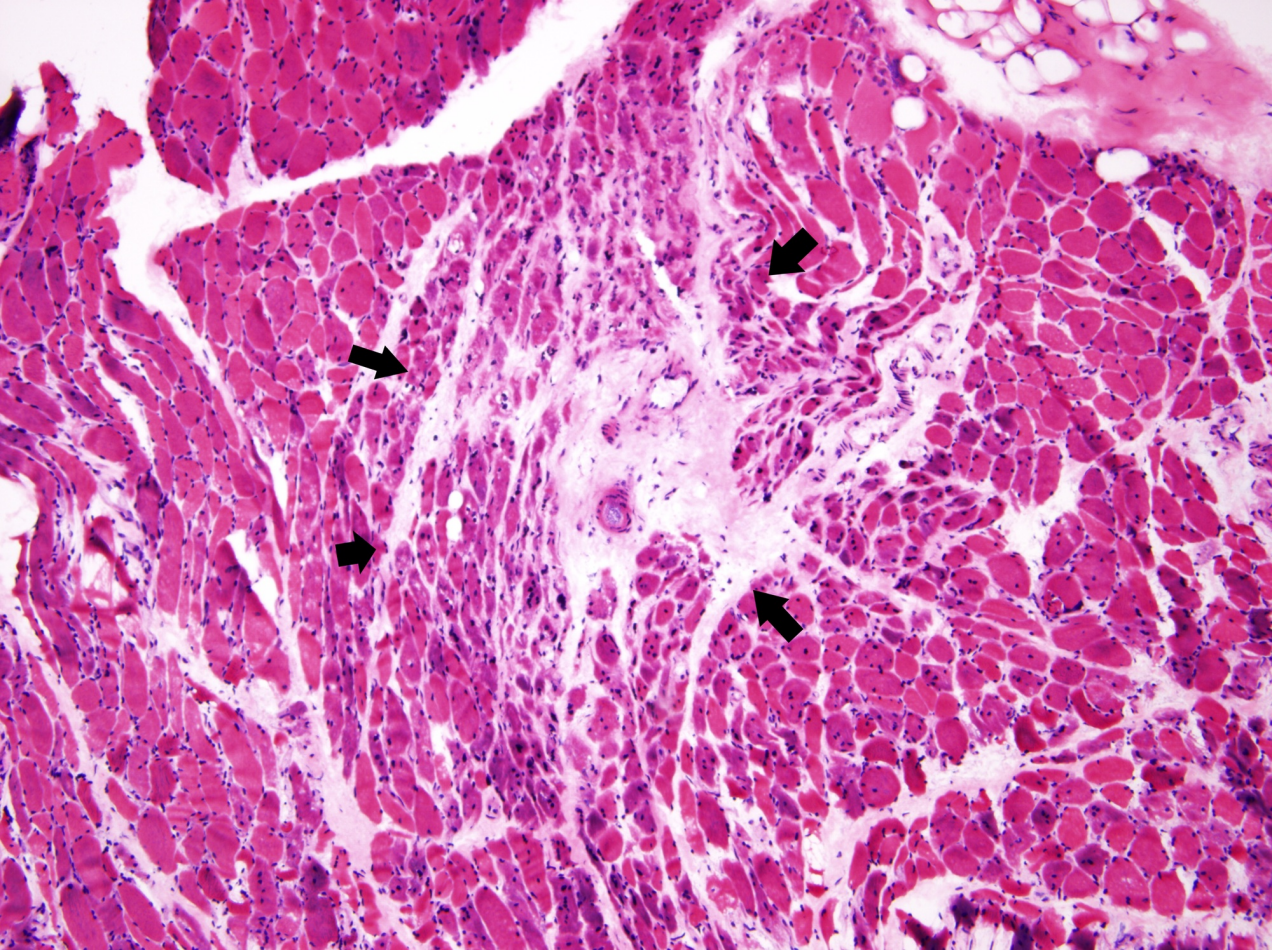
Pathology slide from the muscle biopsy showing perimysial atrophy (present between the arrows)

**Figure 7:**
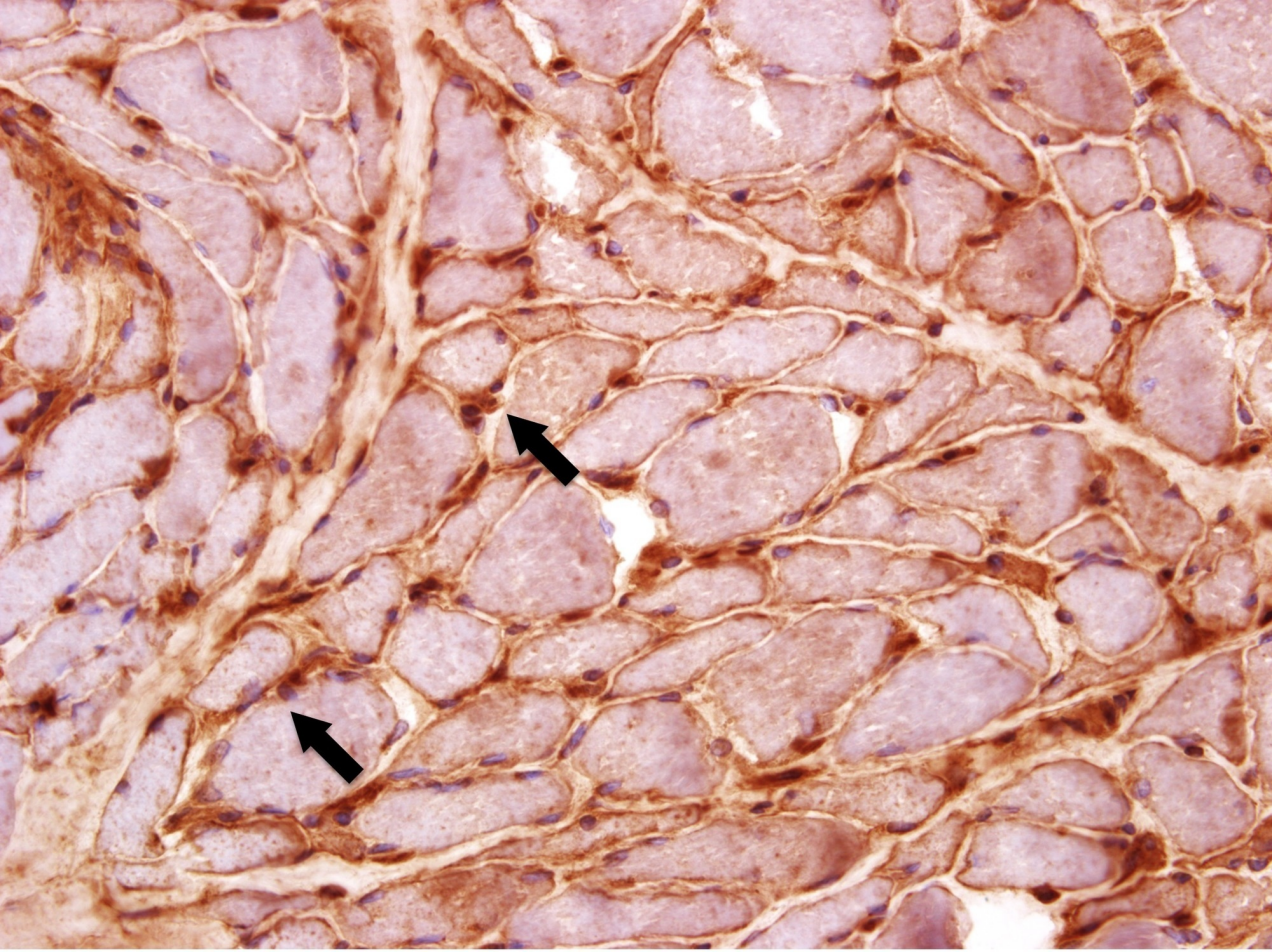
Pathology slides from the muscle biopsy showing the sarcolemmal staining with Class I MHC IHC stain (marked by arrows) MHC: major histocompatibility complex, IHC: immunohistochemistry.

**Figure 8:**
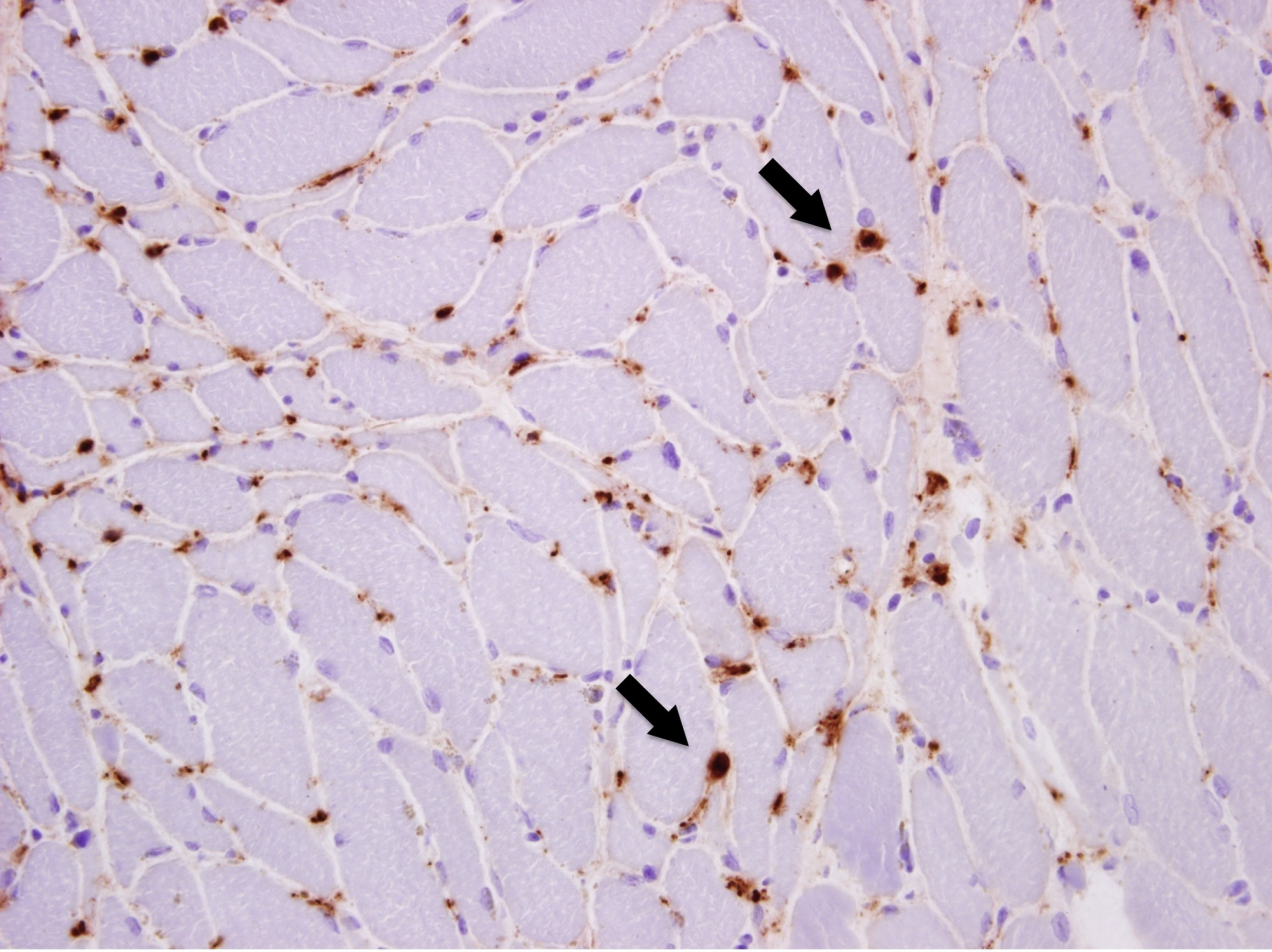
Pathology slides from the muscle biopsy showing capillary staining with complemental C5b-9 IHC (marked by arrows) IHC: immunohistochemistry.

An ultrasound-guided liver biopsy revealed severe diffuse macrovesicular hepatic steatohepatitis involving 80%-90% of the liver parenchyma, mild intracanalicular cholestasis, prominent Mallory-Denk bodies within ballooning hepatocytes and bridging fibrosis on trichrome stain (Figures 4-5). Neurologic electrophysiology studies showed normal nerve conduction, fibrillation and positive waves in all muscles and low amplitude motor unit action potentials in the majority of the muscles studied, particularly in the proximal muscles. In summary, it showed electrophysiologic evidence of a diffuse myopathy with features of muscle membrane irritability. A subsequent muscle biopsy revealed atrophic fibers with a perimysial distribution (as seen in Figure 6), increased immunohistochemistry (IHC) labeling for major histocompatibility complex (MHC) 1 (as seen in Figure 7), and capillary complement staining (as seen in Figure 8), suggesting an autoimmune myositis. There were no features to suggest autoimmune hepatitis. Near the end of her hospitalization, she developed confusion, progressive hypoxia, and succumbed to multi-organ dysfunction.

## Discussion

The muscle biopsy features of atrophic myocytes in perifascicular distribution, MHC-1 labeling and capillary complement staining along with an elevated aldolase and normal creatine kinase and juxta-articular osteopenia seen on hand X-ray make the diagnosis of inflammatory myopathy with perimysial pathology (IMPP). IMPP is a subtype of an inflammatory myopathy classically associated with selective elevation of aldolase with pulmonary and joint involvement [3-4]. In a recently published retrospective study of 57 patients with IMPP, dysphagia was present in 57% of the patients and a positive ANA and Jo-1 antibodies were noted in only 51% and 37% of the patients [4].

As no etiology of liver enzyme elevation was evident after a thorough history and physical examination and review of laboratory results, liver biopsy was pursued and it revealed diffuse steatohepatitis and prominent fibrosis. The patient did not have any major risk factors for metabolic syndrome such as obesity or metabolic syndrome [5] and her liver enzymes elevation persisted in spite of tremendous weight loss, which is the treatment for NAFLD associated with metabolic syndrome [1]. Rapid weight loss from starvation is a known cause of secondary NAFLD and our patient had decreased caloric intake due to dysphagia and evidence of malnutrition (cachexia, low albumin, and serum creatinine). Therefore, starvation-induced steatosis is the most likely etiology of her steatohepatitis. Secondary causes of NAFLD should be considered in patients who do not fit the clinical phenotype of primary NAFLD. The most common secondary causes of NAFLD are medications [6], celiac disease [7], total parenteral nutrition [8], post-surgical weight loss [9], starvation steatosis [10], Wilson’s disease [11] and hepatitis C infection [12]. Rare genetic causes of lipid metabolism are also associated with hepatic steatohepatitis, such as abetalipoproteinemia [13].

Starvation-induced steatosis is classically noted in children with protein-energy malnutrition [14] and is a result of multiple pathogenic sequelae such as increased levels of fatty acids being taken up the liver [10], decreased mobilization from the liver due to impaired very low-density lipoprotein synthesis [15] and decreased fatty acid oxidation from loss of peroxisomes and impaired function of the hepatic mitochondria [16]. In adults, starvation-induced steatosis has been reported in patients who experience weight loss after pancreaticoduodenectomy and war prisoners [17-19]. Sim et al. described a patient who had a similar progression of a normal liver to decompensated cirrhosis from NAFLD in the context of rapid post-surgical weight loss after a pancreaticoduodenectomy within six months of surgery [18]. The delay in diagnosis of the myositis could have contributed significantly to the unfavorable outcome in this case. IMPP is usually responsive to corticosteroid treatment [4] but our patient did not receive appropriate therapy until she was in respiratory failure from recurrent aspiration.

## Conclusions

In summary, we describe an unusual case of starvation-induced steatosis, which arose secondary to IMPP, a rare form of myositis. This case highlights two important points. The secondary causes of NAFLD should be entertained in a patient without classical risk factors of primary NAFLD and a normal chronic liver disease workup. Secondly, normal CK and elevated aldolase in a patient with myositis could indicate a diagnosis of IMPP.
